# Clinical Feasibility of a Just-in-Time Adaptive Intervention App (iREST) as a Behavioral Sleep Treatment in a Military Population: Feasibility Comparative Effectiveness Study

**DOI:** 10.2196/10124

**Published:** 2018-12-07

**Authors:** I Wayan Pulantara, Bambang Parmanto, Anne Germain

**Affiliations:** 1 Health and Rehabilitation Informatics Laboratory Department of Health Information Management University of Pittsburgh Pittsburgh, PA United States; 2 Sleep and Behavioral Neuroscience Center Department of Psychiatry University of Pittsburgh Pittsburgh, PA United States

**Keywords:** just-in-time adaptive intervention, insomnia, sleep, mHealth, mobile health, interactive Resilience Enhancing Sleep Tactics (iREST), behavioral therapy, brief behavioral therapy for insomnia, cognitive behavioral therapy for insomnia

## Abstract

**Background:**

Although evidence-based cognitive behavioral sleep treatments have been shown to be safe and effective, these treatments have limited scalability. Mobile health tools can address this scalability challenge. iREST, or interactive Resilience Enhancing Sleep Tactics, is a mobile health platform designed to provide a just-in-time adaptive intervention (JITAI) in the assessment, monitoring, and delivery of evidence-based sleep recommendations in a scalable and personalized manner. The platform includes a mobile phone–based patient app linked to a clinician portal.

**Objective:**

The first aim of the pilot study was to evaluate the effectiveness of JITAI using the iREST platform for delivering evidence-based sleep interventions in a sample of military service members and veterans. The second aim was to explore the potential effectiveness of this treatment delivery form relative to habitual in-person delivery.

**Methods:**

In this pilot study, military service members and veterans between the ages of 18 and 60 years who reported clinically significant service-related sleep disturbances were enrolled as participants. Participants were asked to use iREST for a period of 4 to 6 weeks during which time they completed a daily sleep/wake diary. Through the clinician portal, trained clinicians offered recommendations consistent with evidence-based behavioral sleep treatments on weeks 2 through 4. To explore potential effectiveness, self-report measures were used, including the Insomnia Severity Index (ISI), the Pittsburgh Sleep Quality Index (PSQI), and the PSQI Addendum for Posttraumatic Stress Disorder.

**Results:**

A total of 27 participants completed the posttreatment assessments. Between pre- and postintervention, clinically and statistically significant improvements in primary and secondary outcomes were detected (eg, a mean reduction on the ISI of 9.96, *t*_26_=9.99, *P*<.001). At posttreatment, 70% (19/27) of participants met the criteria for treatment response and 59% (16/27) achieved remission. Comparing these response and remission rates with previously published results for in-person trials showed no significant differences.

**Conclusion:**

Participants who received evidence-based recommendations from their assigned clinicians through the iREST platform showed clinically significant improvements in insomnia severity, overall sleep quality, and disruptive nocturnal disturbances. These findings are promising, and a larger noninferiority clinical trial is warranted.

## Introduction

Sleep disturbances such as insomnia and nightmares are among the most prevalent complaints reported by post–9/11 military service members (SMs) and veterans [1-3]. Insomnia affects between 40% and 70% of SMs and veterans [4] and can compromise readiness by impairing critical cognitive and moral reasoning abilities while increasing the risk of injuries and costly mishaps due to the resulting fatigue [5].

Insomnia also constitutes a robust risk factor for poor psychological health outcomes, including posttraumatic stress disorder (PTSD), major depressive disorder, suicidal tendencies, hazardous alcohol use, and addictive disorders [6-8]. Furthermore, insomnia impedes the response to treatment of those aforementioned conditions and increases the risk of onset or recurrence [9].

Insomnia is a treatable sleep disorder and a modifiable risk factor of compromised readiness and health. The National Institutes of Health and the American College of Physicians recommend nonpharmacological treatments for insomnia [10,11]**.** Nonpharmacological treatments are commonly the core of cognitive behavioral therapy for insomnia (CBTI). These treatment protocols are typically delivered in person over 1 to 4 sessions (brief version) or to 5 to 8 sessions (standard version) and are usually delivered by a licensed psychologist trained in behavioral sleep medicine [12-14] or a master’s level clinician [15-17]. CBTI has been shown to be safe, effective, and associated with durable improvements [18] in the general population [19-21] and in military samples [22-25].

Nevertheless, the scalability of CBT for sleep disturbances remains limited. One of the main barriers in making CBTI widely available is the shortage of trained clinicians and availability of expertise outside urban centers. For instance, specialty sleep care clinics are not readily available in rural areas in the United States, where approximately 25% of veterans are located [26], and the more than 150 countries where US Armed Forces are stationed. Furthermore, the traditional in-person treatment format often creates barriers to receiving or adhering to treatment visit schedules due to travel distance (to and from the clinics), conflict with work and family schedules, childcare availability, etc. To reduce these potential burdens, brief behavioral treatment protocols (1 to 4 sessions) and online programs have been developed and tested. Brief in-person programs yield comparable or greater benefits as standard longer 6- to 8-week CBTI protocols [27-31]. Telehealth programs [32] and online commercial treatment programs such as SHUTi (BeHealth Solutions LLC) [33,34], Sleepio (Sleepio Ltd) [35], and RESTore (CCBT Ltd) [36] have also been shown to be efficacious and typically require anywhere between 5 weeks to several months of patient engagement [25,33,37,38]. From the patient perspective, traditional CBTI typically requires them to keep a paper sleep diary, which is cumbersome. Most importantly, the rigid schedule of the current CBTI delivery formats (ie, weekly in-person visits) limits the clinician’s ability to personalize the intervention (ie, deliver the right intervention, at the right time, to the right patient).

**Figure 1:**
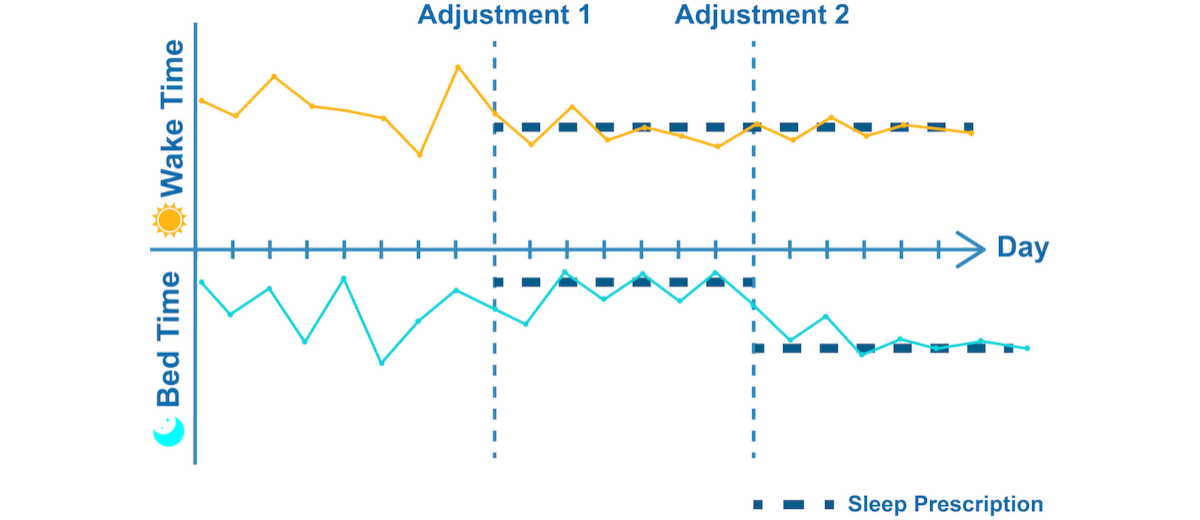
An illustration of just-in-time adjustment of sleep recommendations consistent with sleep restriction and stimulus control based on changes in a patient’s sleep pattern. Before the start of the treatment, a high night-to-night variability in wake times and bedtimes was observed. A sleep restriction recommendation was sent to the patient (Adjustment 1). After several nights, the patient adjusted to this restriction and achieved a reduction in the night-to-night wake/bedtimes variability. At this point, the interactive Resilience Enhancing Sleep Tactics (iREST) portal would suggest a reduction in the amount of sleep restriction (lengthening the recommended time allowed in bed). With clinician approval, this recommendation was sent to the patient’s iREST app (Adjustment 2).

Current advancements in mobile technology and increases in its adoption have the potential to increase access to evidence-based behavioral sleep treatments as well as to enhance the efficacy of these interventions by tailoring them to each individual’s dynamic moment-to-moment needs. For example, a patient with insomnia typically shows high night-to-night variability in wake times and bedtimes, which leads to irregular sleep duration and unpredictable sleep quality (Figure 1).

Baseline bedtime and wake times are determined by data collected by the patients using their mobile phone. Based on these parameters, a clinician is likely to use principles of sleep restriction [39] and/or stimulus control [40]. Sleep restriction is one of the most common behavioral treatments for insomnia and aims to optimize the predictability and quality of sleep by implementing regular wake times and bedtimes. Stimulus control aims to reinforce learned associations between sleep and specific environmental cues (eg, bed, bedroom). Based on data collected with daily sleep/wake diaries, the interactive Resilience Enhancing Sleep Tactics (iREST) portal calculates and suggests personalized recommendations for the implementation of sleep restriction and stimulus control. The clinician can then review and approve or modify these recommendations (or prescriptions) before they are forwarded to the patient through the iREST app (Figure 1, Adjustment 1). Recommendations sent to a patient include specific information on what new sleep behaviors should be adopted, how to implement the recommendations, and the rationale supporting each recommendation. As the patient adopts these recommendations, changes in behaviors and improvements in sleep quality and predictability are detected by the iREST system (Figure 1, Adjustment 2), which iteratively reassesses what behavioral changes may be required and, again with the clinician’s approval, sends an adjustment in the personalized recommendations in a just-in-time fashion [41] until the desired sleep outcomes are achieved (ie, regular sleep behaviors and satisfactory sleep quality). This sort of adaptability includes personalization of the intervention not only at the beginning of the episode of care but throughout the intervention period, in the form of frequent iterative adjustments based on patient-reported data. This type of adaptability and personalization in delivering evidence-based interventions is known as a just-in-time adaptive intervention (JITAI) [42].

**Figure 2:**
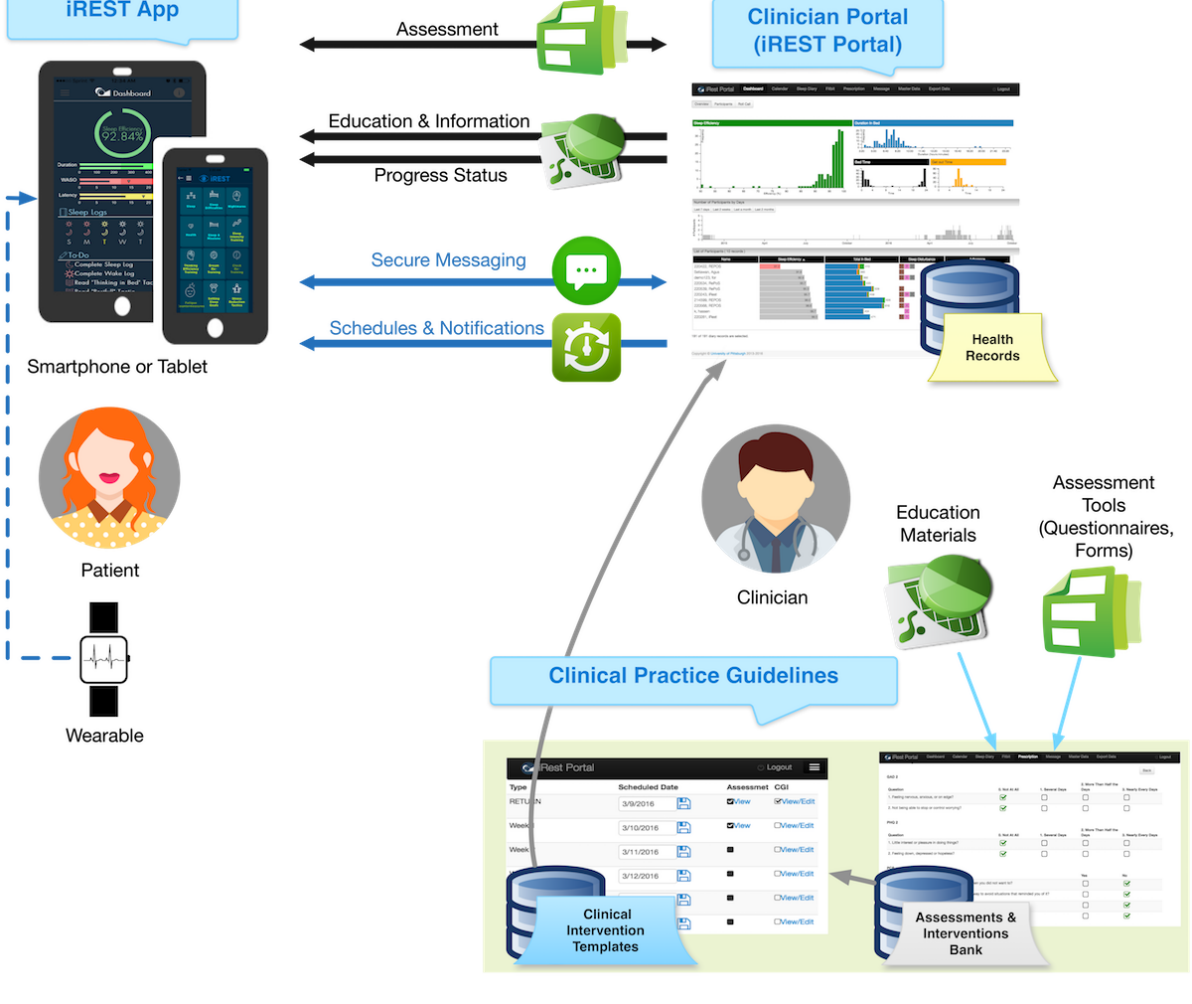
A model representing the interactive Resilience Enhancing Sleep Tactics (iREST) app and clinician portal’s two-way interactions including assessment, education/information delivery, progress reporting, scheduling, notification delivery, and secure messaging. The model also shows objective data gathering using wearable devices.

With this in mind, we developed iREST [43]. iREST is a JITAI implementation of existing behavioral sleep intervention techniques, particularly the military-version brief behavioral therapy for insomnia [17], an intervention that has been found to be effective in SMs and veterans. The iREST system consists of the following (Figure 2):

- Cross-platform mobile phone app [43] that records sleep data, shows feedback and related educational materials, and provides cues and notifications
- Web-based portal that allows therapists to monitor sleep information, prescribe treatment, and engage participants via secure messaging
- Wearable integration that allows objective measurement of the patient’s sleep-wake pattern. The preliminary feasibility report on this integration has been published elsewhere [43]
- Communication protocol that allows real-time bidirectional exchange of data among the app, portal, and wearable sensors

iREST, as a mobile phone–based intervention, has the potential to improve the delivery of traditional CBTI with such novel features as personalization and context awareness. Assessments and interventions are best delivered when they are personalized to fit each individual’s needs and conditions [44,45]. iREST can further tailor the treatment by dynamically adapting both the assessment and intervention. This ability to adapt the intervention can expand to accommodating the environment and social situation, especially important for the military population where training or deployment may not be compatible with prescribed sleep treatments. Such continual adaptation requires personalization of the intervention not only at the beginning of the episode of care but also frequent iterative adjustments during the course of care—something for which a JITAI such as iREST may reveal promising potential.

This pilot study first sought to evaluate the potential effectiveness of digital monitoring and delivery of evidence-based CBT for sleep disturbances in this sample using an open-trial design. In addition, to provide a comparative effectiveness framework, results were compared with previously published effect sizes and rates of treatment responses and remission following traditional [46] in-person CBT for sleep disturbances in this population [17].

## Methods

### Participants

The University of Pittsburgh Institutional Review Board approved this study. SMs and veterans between the ages of 18 and 60 years were recruited from other studies (Military Operational Medicine Research Program proposal number PT130572, PI: Reifman/Germain; Military Operational Medicine Research Program log number 11293006, PI: Germain; log number 13154004, PI: Okonkwo) that used postcards, flyers, study websites, social media/Facebook (San Francisco, CA), and public television advertisements for recruiting purposes. Since our study required participants to use their own device, eligible SMs and veterans had to both own a mobile phone with internet access and be fluent in the use of that mobile phone. Other eligibility criteria included the presence of a clinically significant sleep complaint as determined by a baseline score of 10 or higher on the Insomnia Severity Index (ISI) [47] and having consistently experienced sleep disturbances for at least 1 month. Participants who were diagnosed with obstructive sleep apnea or who scored greater than or equal to 4 on the STOP-BANG (snoring, tiredness, observed apnea, blood pressure, body mass index, age, neck size, and gender) questionnaire [48-50] were excluded from the study. Other exclusion criteria included a history of psychotic disorder or bipolar disorder, the presence of symptoms of narcolepsy or any other sleep disorder requiring further evaluation and treatment, the presence of any severe or untreated psychiatric disorders associated with marked impairments in functioning, and any scheduled/imminent military deployment during the course of the study. Finally, pregnant or breastfeeding women were not included in the study.

### Screening Procedures

After obtaining each participant’s verbal consent, a telephone screening was conducted to assess eligibility prior to the initial in-person visit. Screening questions were related to the current use of a mobile phone, past and current psychiatric and physical health, and the presence of any suspected or diagnosed physiological sleep disorders or sleep apnea. Eligible participants were invited for an in-person consent and assessment visit.

After obtaining written informed consent, participants underwent a 2-part diagnostic evaluation that included a diagnostic interview and a series of screening questionnaires. The diagnostic interview focused on assessing the insomnia, presence and severity of trauma history, alcohol/substance use disorders, other psychiatric disorders, and current physical health. A weekly consensus meeting was held to review diagnostic information and establish the participant eligibility for the open-trial phase of the study. Participants also completed a series of self-report screening questionnaires:

- Structured Clinical Interview for the *Diagnostic and Statistical Manual of Mental Disorders, Fourth Edition* (DSM-IV), nonpatient version [51]: used to assess the participant’s past and current psychiatric history
- DSM Sleep Disorder: developed locally and similar to the Structured Clinical Interview, this instrument assesses the presence of core symptoms of sleep disorders as defined by the International Classification of Sleep Disorders [52], including insomnia, sleep-disordered breathing, restless legs syndrome and other sleep-related movement disorders, and parasomnias
- PTSD Checklist–Civilian version (PCL-C) [53]: used to measure PTSD symptoms; only participants with PCL-C less than 51 were included in the study
- STOP-BANG [54]: a set of 8 yes/no questions performed to assess the participant’s risk for developing sleep apnea
- Insomnia Severity Index (ISI) [47]: used to assess the subjective severity of participant’s insomnia symptoms
- Pittsburgh Sleep Quality Index (PSQI) [55]: administered to assess different components of a participant’s sleep quality (cutoff of 5 differentiating between good and bad sleepers)
- PSQI Addendum for PTSD (PSQI-A) [56,57]: performed to assess the frequency of disruptive nocturnal behaviors commonly experienced by trauma-exposed individuals
- Epworth Sleepiness Scale (ESS) [58]: used to assess a participant’s daytime sleepiness, with higher scores indicating greater sleepiness

### Outcome Measures

Because insomnia is the most prevalent sleep disorder among post–9/11 SMs and veterans [59,60], the ISI [47] was used as the primary sleep outcome metric. The ISI is a 7-item self-administered questionnaire that subjectively assesses the severity of a participant’s insomnia symptoms, including level of satisfaction with sleep, noticeability and extent of daytime impairment, and additional concerns caused by sleep problems. Each item has a scale with a range from 0 to 4, with a total score of 10 or higher reflecting the presence of clinically significant insomnia [47]. Daytime sleepiness was assessed using the ESS [58], an 8-item self-report questionnaire where respondents are asked to rate, on a scale from 0 to 4, their usual chances of dozing off or falling asleep while engaged in 8 different activities. The overall ESS score can range from 0 to 24, where a score of 10 or higher indicates clinically significant somnolence.

Overall sleep quality was assessed using the PSQI [55] and PSQI-A [56,57]. The PSQI is an 18-item self-administered questionnaire that assesses different components of sleep quality with scores ranging from 0 to 21. A score of 5 or higher has been shown to reflect clinically significant sleep complaints. Disruptive nocturnal behaviors were assessed using the PSQI-A, which is a 7-item self-report measure that assesses the severity of 7 disruptive nocturnal behaviors commonly experienced by trauma-exposed individuals [56,57]. PSQI-A scores range from 0 to 21, with a score of 4 or higher indicating clinically significant disruptive nocturnal behaviors.

Given the common comorbidity between sleep disturbances and psychiatric symptoms, participants also completed the PCL-C [53], the Patient Health Questionnaire 9-item (PHQ-9) [61] to measure symptoms of depression, and the Generalized Anxiety Disorder 7-item (GAD-7) [62] to measure symptoms of anxiety. The PCL-C is a 17-item self-report rating scale of PTSD symptom severity, with higher scores reflecting more severe symptomatology. The PHQ-9 item assesses the frequency of 9 symptoms of depression over the preceding 2 weeks. Scores of 5, 10, 15, and 20 represent no to mild, moderate, and moderately severe depression, respectively. Finally, the GAD-7 is a brief self-report measure of symptoms of generalized anxiety.

Consistent with the previous trials [17,46], treatment response was defined as a reduction of 8 or more points on the ISI [63]. Furthermore, remission was defined as meeting the treatment response criteria and achieving a posttreatment ISI score below the clinical threshold of 7. Treatment response was also assessed with more global measures of improvements, using the PSQI (defined as a decrease of at least 3 points from pre- to posttreatment) [21,46] and the Patient- and Clinician-Rated Clinical Global Improvement Scales [64-66].

### Exploratory Evaluation of Noninferiority

The second aim of the study was to compare clinical improvements in sleep and psychiatric symptoms using iREST relative to habitual, in-person delivery formats of evidence-based behavioral sleep treatments. To do so, we extracted data from 2 previously published trials [17,46]. The first trial included an 8-week treatment arm (CBTI+IRT) that combined in-person CBTI and imagery rehearsal therapy (IRT) for nightmares [46]. The second trial tested an abbreviated, 4-week CBTI protocol specifically designed for SMs and veterans [17]. In this trial, the intervention was delivered during a 45-minute session in week 1 followed by a booster telephone session in week 3 [67]. Both of these trials enrolled SMs and veterans presenting chronic, service-related sleep complaints and employed the same sleep measures (ie, ISI, PSQI, PSQI-A, and ESS). Symptoms of PTSD were assessed with the PCL-C. Symptoms of depression and anxiety were assessed with the Beck Depression Inventory [68] and the Beck Anxiety Inventory (BAI) [69].

**Figure 3:**
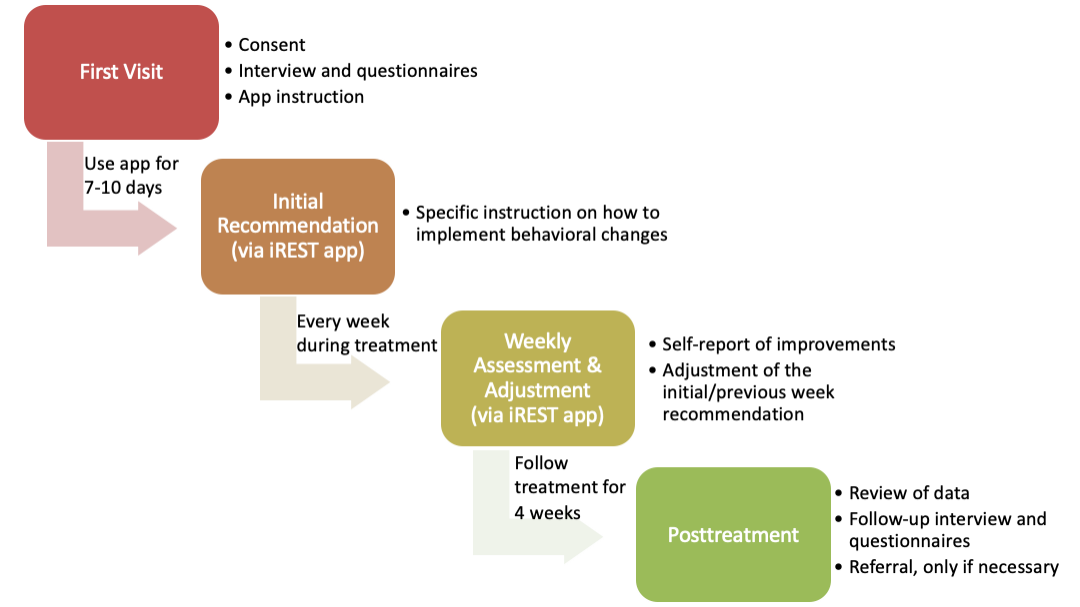
The interactive Resilience Enhancing Sleep Tactics (iREST) study workflow.

### Treatment Conditions

Figure 3 depicts this study’s overall workflow. After providing their written informed consent, participants were issued a 6-digit ID and then asked to complete the series of self-report questionnaires aimed at assessing their military history and any demographic variables, baseline sleep quality, current sleep habits and behaviors, current psychological well-being, and overall perceived physical health. Participants also completed clinician-administered interviews to assess the presence of psychiatric disorders. After the interviews, participants downloaded the iREST app on their personal mobile phone and received instructions on how to use the app. They were instructed to complete the morning and evening sleep diary for the next 7 to 10 days, at which point they would receive their personalized sleep recommendations via the app. They were also instructed to contact their clinicians via the text messaging function or by telephone as needed. This first visit took approximately 90 minutes.

After this initial period of 7 to 10 days with the app, participants received their individualized recommendations via the app, with specific instructions on how to implement recommended behavioral changes. Each week, they completed a short battery of self-report measures to assess overall perceived improvements in sleep, side effects, and symptoms of depression, anxiety, and PTSD to monitor progress. Clinicians reviewed participant sleep information and reported symptoms and improvements via the portal. Adjustments to the initial recommendations were provided on average on a weekly basis. During the intervention phase, participants who reported an exacerbation of symptoms were scheduled for telephone or in-person visit. After the intervention phase, participants who continued to experience significant sleep complaints were offered in-person sleep consults with the clinician and/or referral to a sleep clinic or mental health services.

### Statistical Analysis

Descriptive statistics were performed to describe the demographic characteristics of the study participants using frequencies for categorical variables and means and standard deviations for continuously measured demographic variables.

SPSS Statistics software version 24.0 (IBM Corp) was used to assess pre- to postintervention changes in sleep and psychiatric symptom severity. For the first aim, paired *t* tests were used to test pre- to posttreatment differences on self-reported sleep and psychiatric symptom measures. To better contextualize the magnitude of improvement, Cohen *d* effect sizes were also computed. In addition, mixed model analyses of variance (ANOVAs) were performed to explore whether the improvement in outcomes differed based on the presence or absence of comorbid disorders.

For the second aim, descriptive statistics were performed to describe the demographic characteristics of the study participants using frequencies for categorical variables and means and standard deviations for continuously measured demographic variables. Using a chi-square test for categorical variables and ANOVAs for continuous variables, each demographic variable was compared with the same variable from the previously published in-person trials [17,46] to determine whether there existed any statistically significant differences between the distribution in this study sample and the previous one. Rates of treatment response and remission across 3 delivery formats were compared using the chi-square test. Finally, a mixed model ANOVA was conducted on the primary sleep outcome (ISI) to explore on whether there were different effects between the groups.

## Results

### Participant Flow

**Figure 4:**
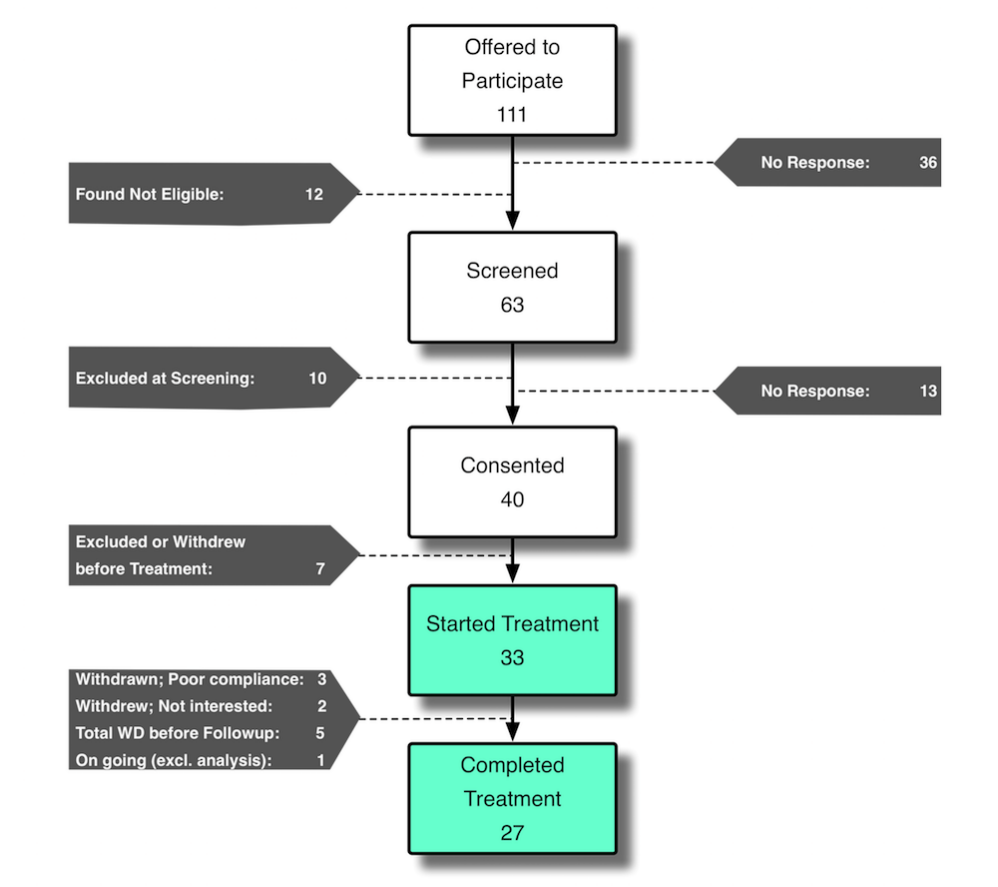
Participant flow diagram.

A total of 111 SMs and veterans expressed interest in participating in this pilot feasibility study (Figure 4). Of these, 40 provided written consent and 33 were found eligible to complete the study. Out of the participants who started the intervention, 84% (27/33) completed the posttreatment/follow-up assessment and were included in the follow-up analysis.

### Demographics

Of the 5 participants who did not finish the treatment, 3 were excluded due to poor compliance and 2 withdrew because they were no longer interested in the study. There were no significant differences between those who completed the study and those who did not. Table 1 shows the demographics and baseline scores for the 27 completers.

### Pre- to Posttreatment Changes in Sleep and Psychiatric Symptoms

The pre- and postintervention tests show statistically significant improvement in primary and secondary sleep outcomes. As shown in Table 2, the mean reduction on the ISI was 9.96, *t*_26_=9.99, *P*<.001, which reflects a decrease by at least 1 severity category on this measure. Additionally, there was marked improvement in sleep quality, with a mean reduction on the PSQI of 6.67, *t*_26_=8.22, *P*<.001, and mean reduction on the overall severity of disruptive nocturnal disturbances on the PSQI-A of 2.37, *t*_26_=3.55, *P*=.001. Finally, there was a decrease in daytime sleepiness, with a mean reduction on the ESS of 2.04, *t*_26_=2.98, *P*=.006. Clinically and statistically significant improvements in symptoms of depression, anxiety, and PTSD were also detected (see Table 2 for details on these improvements).

**Table 1:** Participant demographics and baseline scores (N=27).

Variable	Value
Male, n (%)	24 (89)
White, n (%)	20 (74)
Age (years), mean (SD)	36.48 (9.64)
Army, n (%)	15 (56)
Current posttraumatic stress disorder, n (%)	8 (30)
Using psychotropic medications, n (%)	7 (26)
Current mood or anxiety disorder, n (%)	10 (37)

**Table 2:** Mean score changes pre- and postintervention.

Variable	Baseline score, mean (SD)	Posttreatment score, mean (SD)	Mean change (SE)	*t* statistics (*df*)	*P* value	Cohen *d* effect sizes
ISI^a^	15.59 (4.13)	5.63 (4.76)	9.96 (1.00)	9.99 (26)	<.001	1.93
ESS^b^	7.07 (4.51)	5.04 (3.82)	2.04 (0.69)	2.98 (26)	.006	0.57
PSQI^c^	11.81 (3.19)	5.15 (3.43)	6.67 (0.81)	8.22 (26)	<.001	1.58
PSQI-A^d^	4.59 (3.83)	2.22 (2.81)	2.37 (0.67)	3.55 (26)	.001	0.71
PCL-C^e,f,g^	38.41 (14.10)	27.22 (11.87)	11.19 (1.86)	6.58 (26)	<.001	1.19
PHQ-9^h^	8.41 (5.22)	3.63 (5.34)	4.78 (0.81)	5.89 (26)	<.001	1.13
GAD-7^i^	6.17 (5.32)	2.91 (2.94)	3.26 (0.90)	3.64 (22)	.001	0.74

^a^ISI: Insomnia Severity Index.

^b^ESS: Epworth Sleepiness Scale.

^c^PSQI: Pittsburgh Sleep Quality Index.

^d^PSQI-A: Pittsburgh Sleep Quality Index–Addendum for Posttraumatic Stress Disorder.

^e^PCL-C: Posttraumatic Stress Disorder Checklist–Civilian.

^f^PCL-C scores were not normally distributed and a natural log transformation was used in the analyses.

^g^Raw scores are presented.

^h^PHQ-9: Patient Health Questionnaire 9-itm.

^i^GAD-7: Generalized Anxiety Disorder 7-item.

**Table 3:** Insomnia improvement grouped by comorbidity diagnoses.

Grouping variable and effect		*F*	*P* value
**Posttraumatic stress disorder diagnosis**			
	Time	84.50	<.001
	Group	0.22	.64
	Time × group	0.25	.62
**Mood and anxiety diagnosis**			
	Time	87.86	<.001
	Group	3.01	.09
	Time × group	0.25	.62

### Rates of Treatment Response and Remission With the Interactive Resilience Enhancing Sleep Tactics App

Using the global measures of clinical improvement, 74% (20/27) of participants reported that they were much or very much improved posttreatment, whereas clinicians rated 82% (22/27) of participants much or very much improved posttreatment. Using the criterion of a decrease of at least 3 points on the PSQI, 85% (23/27) of patients showed improvements in global sleep quality and, of those, 19 achieved full remission, defined as a posttreatment PSQI score of less than 5. Using the stringent definition of treatment response of a reduction by 8 points or more on the ISI, 70% (19/27) of participants met the criterion for treatment response and 16 presented full remission of insomnia (ie, ISI score less than 7 posttreatment—59% (16/27) of the full sample and 84% (16/19) of responders).

### Exploratory Assessment of Noninferiority of the Interactive Resilience Enhancing Sleep Tactics App Relative to Standard and Abbreviated Cognitive Behavioral Therapy for Insomnia

As shown in Table 4, there were no statistically significant demographic differences between participants in this study (iREST) and those in the traditional trials used as a control. Furthermore, participants for the iREST study and prior traditional studies were drawn from the same geographical area. For all 3 studies, participants had to be able to attend an in-person assessment at the University of Pittsburgh Military Sleep Tactics and Resilience Research Team office.

Mixed model ANOVAs were also performed within-group to explore whether the improvement in outcomes differed based on the presence or absence of comorbid PTSD and mood and anxiety disorders. After controlling for baseline ISI scores, the only significant effect observed was expressed in terms of time (pre- to post-). No significant effect from comorbidity conditions or any significant interactions between time and these conditions was observed. See Table 3 for additional results.

**Figure 5:**
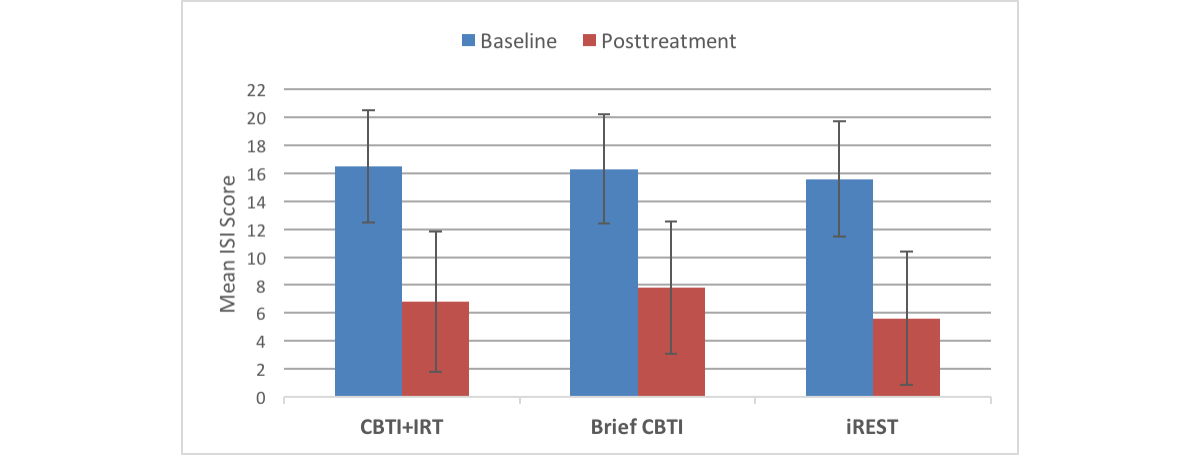
Reductions in the Insomnia Severity Index from baseline to posttreatment with cognitive behavioral therapy for insomnia (CBTI; 8 in-person visits over 8 weeks), brief CBTI (2 in-person visits over 4 weeks), and the interactive Resilience Enhancing Sleep Tactics (iREST) app (visits=interventions through the app over 4 weeks).

On the ISI, the CBTI+IRT, brief CBTI, and iREST yielded large and clinically significant improvements, with Cohen *d* effect sizes of *d*=1.50, *d*=1.96, and *d*=1.93, respectively (shown in Figure 5). Additionally, significant improvements in sleep quality, or reductions in PSQI score, were also observed in all 3 groups, with *d*=1.45 in the CBTI+IRT group, *d*=1.56 in the brief CBTI group, and *d*=1.58 in the iREST group. There were also significant improvements in PSQI-A scores for the CBTI+IRT and the iREST groups, with *d*=0.87 and *d*=0.71, respectively.

PTSD symptoms were similarly reduced in the CBTI+IRT and iREST groups pre- to posttreatment, with Cohen *d* effect sizes of *d*=1.08 and *d*=1.19, respectively; a lower Cohen *d* effect size of *d*=0.20 was reported on the brief CBTI group. Depression symptom severity was also significantly reduced in all groups, with *d*=0.65 in the CBTI+IRT group and *d*=0.69 in the brief CBTI group compared with *d*=1.13 in the iREST group. For symptoms of anxiety measured with the BAI, pre- to posttreatment changes in both the CBTI+IRT and brief CBTI groups were nonsignificant (*d*=0.08 and *d*=0.14, respectively), whereas the pre- to posttreatment changes in symptoms of generalized anxiety as measured by the GAD-7 were significantly more pronounced in the iREST group (*d*=0.89).

Furthermore, a mixed model ANOVA conducted before and after treatment, with time functioning as a within-subject repeated measure on the primary clinical outcome (ISI), showed no significant group × time interaction (*F*_2,53_=0.36, *P*=.70) and no main effect of group (iREST vs brief CBTI vs CBTI+IRT; *F*_2,53_=1.02, *P*=.37). Instead, only a main effect of time was detected (*F*_1,53_= 140.5, *P*<.001). This further suggests that iREST may be noninferior to the in-person brief CBTI and standard CBTI.

**Table 4:** Demographic and clinical information at baseline compared with in-person standard (8 weeks) [46] and brief (4 weeks) cognitive behavioral therapy for insomnia trials in military samples [17].

Characteristics		iREST^a^ (n=27)	CBTI^b^ + IRT^c^ (n=17)	Brief CBTI (n=20)	Statistics	
					χ^2^	*F*_2,61_
**Variable**						
	Male, n (%)	24 (88.9)	14 (88.9)	19 (95)	1.51	—
	White, n (%)	20 (74.1)	12 (70.6)	14 (70)	0.11	—
	Age (years), mean (SD)	36.48 (9.6)	40.0 (14.1)	40.9 (12.0)	—	0.94
	Army, n (%)	15 (55.6)	NR^d^	16 (80)	3.06	
	Current posttraumatic stress disorder, n (%)	8 (29.6)	7 (41.2)	4 (20)	1.18	—
	Using psychotropic medications, n (%)	7 (25.9)	6 (35.3)	5 (25)	0.59	—
	Current mood or anxiety disorder, n (%)	10 (37.0)	2 (11.8)	2 (10)	4.14	—
**Baseline sleep assessment, mean (SD)**						
	Epworth Sleepiness Scale^e^	7.4 (4.6)	NR	7.3 (4.4)	—	—
	Insomnia Severity Index	17.4 (4.0)	16.5 (4.0)	16.3 (3.9)	—	0.52
	Pittsburgh Sleep Quality Index	11.9 (3.9)	10.3 (2.9)	11.3 (3.5)	—	2.14

^a^iREST: interactive Resilience Enhancing Sleep Tactics.

^b^CBTI: cognitive behavioral therapy for insomnia.

^c^IRT: imagery rehearsal therapy.

^d^NR: value for this category was not reported on the CBTI+IRT study.

^e^*t*_46_=0.08

**Figure 6:**
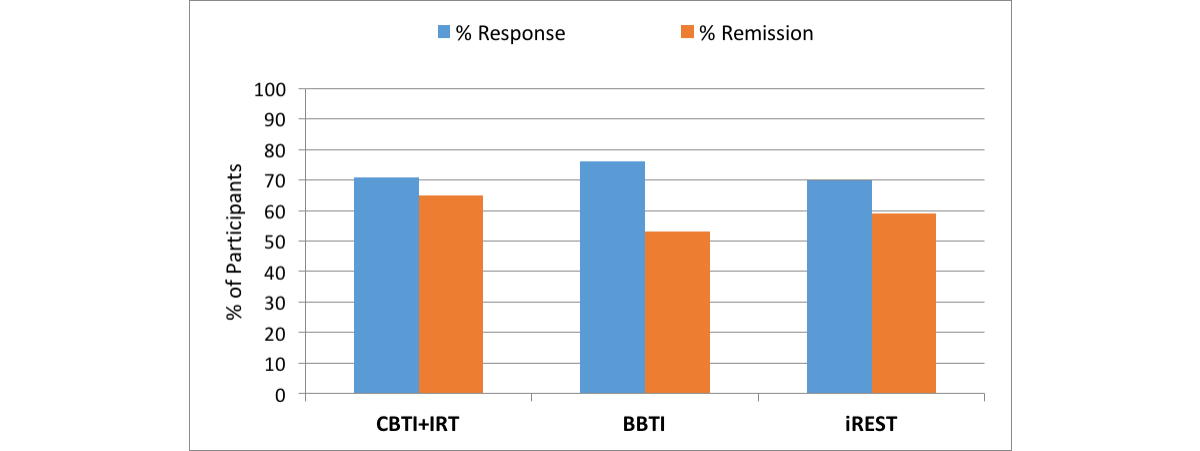
Comparison of the interactive Resilience Enhancing Sleep Tactics (iREST) app and traditional intervention remission and treatment response rates. BBTI: brief behavioral therapy for insomnia; CBTI: cognitive behavioral therapy for insomnia; IRT: imagery rehearsal therapy.

The response rate for the previous in-person brief CBTI study was 76.47% with a remission rate of 52.94%, while the rates for the CBTI+IRT trial were 70.59% and 64.70%, respectively. No significant difference exists between the rates in our trial and the previous in-person brief and standard CBTI studies (illustrated in Figure 6). Here the chi-square values are χ^2^=0.22, *P*=.90, and χ^2^=0.49, *P*=.78, for response and remission, respectively.

## Discussion

### Principal Findings

The purpose of this pilot study was to evaluate clinical feasibility and potential benefits of a novel JITAI app (iREST) for the delivery of evidence-based behavioral sleep treatments. In this study, clinically significant improvements in insomnia, general sleep quality, and disruptive nocturnal behaviors were detected pre- to posttreatment with iREST. Clinically meaningful improvements in symptoms of PTSD, depression, and anxiety were also detected. These findings suggest that iREST, a novel mobile health (mHealth) platform, has a high potential for augmenting the current inventory of evidence-based recommended behavioral sleep treatments.

To explore the potential noninferiority of iREST, we compared clinical outcomes observed with iREST in this study to improvements we observed in previous randomized clinical trials with standard or abbreviated in-person delivery of CBTI [17,46]. Consistent with previous JITAI studies [45,70-73], we found that the implementation of personalized and time-varying JITAI approaches with iREST yielded noninferior outcomes with regard to insomnia or measures of sleep quality and disruptive nocturnal behaviors. The rates of treatment response and remission were also comparable to previously reported rates in CBTI trials [17,46,74-76]. Finally, this exploratory comparison suggested that the magnitude of improvements detected for psychiatric symptoms of PTSD and depression were noninferior with IREST as previously detected in clinical trials. Although improvements in symptoms of anxiety seem to be superior with iREST relative to the standard in-person treatments, the different measures used across trials warrant caution.

The use of a mobile phone app and clinician Web portal, features that allow for real-time monitoring and delivery of personalized treatment prescriptions (eg, bedtime reminders, wake-up alarms, appropriate bibliotherapies, and additional assessments) matching the needs of the individual, contributed to the promising results of this study. Furthermore, delivering the behavioral sleep intervention digitally (through mHealth/mobile app) reduced or eliminated the costs associated with an in-person visit to a sleep clinic (eg, loss of wages and cost for travel, accommodations, and child care). It also potentially addressed access and scalability barriers; through the personalization and prioritization embedded in a JITAI-based system, clinicians could optimize their service so it would reach a greater number of patients while maintaining the same level of care. Last, digital interventions such as JITAI allow seamless integration of patients’ clinical progress and outcomes into the medical center or clinic’s electronic health record system and each patient’s own personal health record. This integration is important in maintaining the continuity of care, especially since insomnia is highly comorbid with other health and psychological conditions [6,77,78].

### Limitations

Inherent to the pilot nature of the study, a first limitation is the relatively small sample size. Therefore, the effect sizes detected in this sample, albeit moderate to large, may be attenuated in a larger, confirmatory noninferiority clinical trial. A second limitation relates to the exclusion of individuals with severe psychiatric disorders or sleep apnea. The high rate of exclusion in this study and previous clinical trials highlights the fact that these disorders are highly prevalent among SMs and veterans [59,60] and hence limit the generalizability of the findings to the more severely affected populations. In a related manner, the inclusion of military SMs and veterans may limit the generalizability of the findings to the general civilian population. Future studies should include a wider set of participants and narrower exclusion criteria to assess the effectiveness and generalizability of the treatment for patients with comorbidities.

### Conclusions

In this preliminary study, iREST, a novel JITAI app, was associated with statistically significant and clinically meaningful improvements in sleep and psychiatric symptoms in a sample of SMs and veterans with chronic, service-related insomnia. Exploratory comparisons strongly suggest that iREST is noninferior to the traditional in-person delivery formats for CBTI for sleep and related psychiatric symptoms. Together, these findings support the notion that iREST and the JITAI approach can be an acceptable and effective approach to enhance the scalability of evidence-based behavioral sleep treatments. Larger confirmatory noninferiority trials are needed in order to fully understand the effectiveness of JITAI-based iREST among military and civilian populations.

## Abbreviations

ANOVA: analysis of variance
BAI: Beck Anxiety Inventory
CBTI: cognitive behavioral therapy for insomnia
DSM-IV: Diagnostic and Statistical Manual of Mental Disorders, Fourth Edition
ESS: Epworth Sleepiness Scale
GAD-7: Generalized Anxiety Disorder 7-item
iREST: interactive Resilience Enhancing Sleep Tactics
IRT: imagery rehearsal therapy
ISI: Insomnia Severity Index
JITAI: just-in-time adaptive intervention
mHealth: mobile health
PCL-C: Posttraumatic Stress Disorder Checklist–Civilian
PHQ-9: Patient Health Questionnaire 9-item
PSQI: Pittsburgh Sleep Quality Index
PSQI-A: Pittsburgh Sleep Quality Index–Addendum for Posttraumatic Stress Disorder
PTSD: posttraumatic stress disorder
SM: service member
STOP-BANG: snoring, tired, observed apneas, blood pressure, body mass index, age, neck size, and gender
